# Plasma and erythrocyte membrane phospholipids and fatty acids in Italian general population and hemodialysis patients

**DOI:** 10.1186/1476-511X-13-54

**Published:** 2014-03-21

**Authors:** Mariarita Dessì, Annalisa Noce, Pierfrancesco Bertucci, Gianluca Noce, Stefano Rizza, Alessandro De Stefano, Simone Manca di Villahermosa, Sergio Bernardini, Antonino De Lorenzo, Nicola Di Daniele

**Affiliations:** 1 Laboratory Medicine, “Tor Vergata” University Hospital, Viale Oxford 81, 00133 Rome, Italy; 2 Department of System Medicine, Nephrology and Hypertension Unit, “Tor Vergata” University Hospital, Viale Oxford 81, 00133 Rome, Italy; 3 Department of System Medicine, Center for Atherosclerosis “Tor Vergata” University Hospital, Viale Oxford 81, 00133 Rome, Italy; 4 Department of Neurosciences, Division of Human Nutrition, University of Rome “Tor Vergata”, I-00133 Rome, Italy

**Keywords:** Fatty acid, Lipid profile, Hemodialysis, ω6/ω3 ratio, Triglycerides

## Abstract

**Background:**

Dyslipidemia and abnormal phospholipid metabolism are frequent in uremic patients and increase their risk of cardiovascular disease (CVD): ω-3 polyunsaturated fatty acids (PUFAs) may reduce this risk in the general population. In this study we compared the plasma and erythrocyte cell membrane composition of PUFAs in a group of Caucasian hemodialysis (HD) patients and in a control group of healthy subjects and evaluated the erythrocyte/cell membrane fatty acid ratio as a marker of the dietary intake of phospholipids. The relationship between ω-3 and ω-6 fatty acids and the possible differences in PUFAs concentrations were also investigated.

**Methods and results:**

After obtaining a fully informed consent, a total of ninety-nine HD patients and 160 non uremic control subjects from “Tor Vergata” University Hospital were enrolled into the study. None of them took antioxidant drugs or dietary supplements for at least 90 days prior to the observation. Blood samples were analysed by gas-chromatographic coupled to a mass spectrometric detector.

The daily intake of total calories, proteins, lipids and carbohydrates is significantly lower in HD patients than in controls (p < 0.001). Most plasma and erythrocyte PUFA were also reduced significantly in HD patients (p < 0.001).

**Conclusions:**

Our results suggest that many classes of PUFAs are lacking in HD patients, due to the removal of nutrients during the dialysis and to persistent malnutrition. A dietary treatment addressed to increase plasma ω-3 PUFAs and to optimize ω-6/ω-3 ratio may exert a protective action and reduce the risk of CVD in HD patient.

## Introduction

Cardiovascular (CV) morbidity and mortality are increased in hemodialysis (HD) patients compared to the general population regardless of gender, age, race and the presence of diabetes mellitus. In particular, CV mortality observed in uremic patients aged 25 to 35 years equals that observed in the general population aged over 85 [1]. The evaluation of these traditional risk factors does not explain the phenomenon completely; it is therefore necessary to examine other factors associated with chronic renal failure such as proteinuria, chronic inflammation, oxidative stress, electrolyte disturbances and malnutrition in particular. However, previous investigations on plasma and erythrocyte concentration of fatty acids in end-stage renal disease (ESRD) patients yielded to contradictory results [2-5].

As kidney function declines, an altered lipid profile is observed in renal patients: total cholesterol, LDL-cholesterol and triglycerides are significantly increased, while HDL-cholesterol is significantly reduced. Low HDL-cholesterol is always associated with hypertriglyceridemia and it is strictly linked to the changes in triglyceride metabolism [6]. Accordingly, Chronic Kidney Disease (CKD) patients show lipid and lipoprotein metabolism disturbances and changes in the metabolism of fatty acids may also develop [7].

Currently, there are no studies designed to investigate the composition of plasma and erythrocyte PUFAs in HD patients in Italy. Aim of the present study was thus to compare plasma and erythrocyte PUFA composition in a group of Italian Caucasians HD patients and in a group of healthy subjects, in order to investigate the presence of a further cardiovascular risk factor in an established high-risk population. The average diet followed during the last twelve months prior to the study was considered. We measured erythrocyte fatty acids as a marker of cell membrane fatty acids as well as of the phospholipid dietary intake.

## Methods

### Study population

The study protocol complied with the Declaration of Helsinki and was appointed by the Ethics Committee of the University Hospital of “Tor Vergata”. Both men and women over the age of 18 years were eligible for the study. A written fully informed consent was provided by all participants before enrollment into the study. The study population consisted of a total of 99 ESRD patients on maintenance HD relating to the Department of “Nephrology and Hypertension” of the University Hospital of “Tor Vergata” (“study group”) and 160 healthy volunteers enrolled at the Department of Occupational Medicine of “Tor Vergata” University (“control group”). ESRD patients received standard bicarbonate hemodialysis with 1.5-2sqm low flux polysulfone membranes for four hours, three times weekly since at least three months. Healthy controls had no history of renal disease, diabetes, hypertension, dyslipidemia, liver disease or cancer. They didn’t take any drug or medication.

### Anthropometry and traditional CV risk profile

The Body Mass Index (BMI) was calculated at enrollment as body weight divided by height squared (kg/m^2^). The post-dialysis body weight (“dry weight”) was considered in HD patients.

Systolic and diastolic blood pressures were registered before dialysis in ESRD patients and at enrollment in healthy controls.

Serum lipid profile components and glucose were determined by The Dimension VISTA 1500 equipment (SIEMENS healthcare Diagnostic, Germany) in all the study population.

### Dietary pattern

We used a questionnaire validated by INRAN (National Research Institute for Food and Nutrition) [8] to investigate the dietary habits of HD patients and control subjects. Frequency of meals and amount of food consumed by the subjects enrolled into the study were evaluated during the twelve months preceding the measurement of plasma and erythrocyte membrane PUFAs. A questionnaire that assessed the eating habits of all subjects enrolled was given to each one to ascertain the frequency of consumption of certain foods for a given period of time (week, month, year). A total of 790 different foods with as many as 67 different nutrients were investigated, not all available for all foods. Shortcomings still exist regarding in particular the vitamins and trace elements. The INRAN_QUEST_SCAI_2005 software developed in ACCESS 2003 was employed for the analysis of the questionnaires.

### Plasma and erythrocyte lipid measurement

Erythrocyte membrane polyunsaturated fatty acid (PUFA) methyl esters were extracted using a modified version of previously reported methods [9-11] and analyzed by a gas-chromatography equipment (GC, model 6890 N, Agilent Technologies Italia Spa), coupled to a mass spectrometric detector (MSD, model Agilent 5973 inert, Agilent Technologies Italia Spa).

### Statistical analysis

Quantitative variables were expressed as means ± standard deviation. Categorical variables were presented as numbers and percentages.

The presence of skewed data was evaluated using visual inspection of Q-Q plots, stem and leaf plots, or box plots and verified using the Shapiro-Wilk test for normal distribution. Unpaired *t* test was employed to compare the data between uremic patients and healthy subjects (controls); non parameter variables were analyzed by Mann–Whitney test.

The significance of the difference between percentages in groups was evaluated using the chi-square test.

The analysis of covariance was used to correct the differences between the variables of interest for age, gender, smoke and BMI. P-value <0.05 was considered statistically significant.

Analysis was performed with the SPSS 15.0 software for Windows platforms, (SPSS, Inc., Chicago, IL).

## Results

The epidemiological and anthropometric features of the whole study population are summarized in Tables 1 and 2. ESRD patients showed a lower total calories daily intake, lower vegetable/animal lipid and protein content, lower carbohydrate, fibers and retinol intake compared to healthy controls (p < 0.0001). Fish intake was not significantly different between the two groups (Table 3).

**Table 1:** Anthropometric and clinical characteristics correlations between HD patients and healthy subjects (controls)

	**HD (n = 99)**	**Controls (n = 160)**	**p**
Age (years)	69.3 ± 14.7	61.3 ± 7.1	<0.01
Gender (male %)	59.6	100	<0.01
BMI (kg/m^2^)	23.0 ± 1.2	22.1 ± 1.5	<0.01
Fasting glucose (mg/dl)	125.2 ± 51.6	91.3 ± 19.3	<0.01
Blood urea nitrogen (before dialysis)	127.6 ± 43.4	-	n.a
Blood urea nitrogen (after dialysis)	39.8 ± 16.7	-	n.a.
Creatinin (mg/dl)	8.0 ± 2.8	0.9 ± 0.4	<0.01
Dialytic vintage (months)	65.8 ± 64.8	-	n.a
Smoking (%)	15.1	5.0	<0.01
Diabetes mellitus (%)	23.2	0	n.a.
Hypertension (%)	79.8	0	n.a.
C-reactive protein	2.4 ± 5.5	1.4 ± 2.7	<0.01

P-Value <0.05 is statistically significant.

HD: hemodialysis. BMI: body mass index n.a.: not applicable.

**Table 2:** Epidemiologic data of the study population

	**HD**	**Controls**
**Primary causes of end-stage renal disease**		
Nephroangiosclerosis (%)	39.4	-
Chronic glomerulonephritis (%)	31.3	-
ADPKD (%)	17.2	-
Chronic pyelonephritis (%)	12.1	-
**Drug therapy**		
Statins (%)	0	0
Antihypertensive drugs (%)	79.8	0
Anticoagulants (%)	65.6	0

HD: hemodialysis, ADPKD: Autosomal dominant polycystic kidney disease.

**Table 3:** Comparison of the daily dietary consumption in HD patients versus the control group

**Daily dietary composition**	**HD (n = 99)**	**Controls (n = 160)**	**P-value**
Kcal (kcal)	1204.0 ± 275.8	1902.0 ± 280.8	<0.001
Vegetable protein (g)	26.8 ± 6.5	62.3 ± 7.5	<0.001
Animal protein (g)	34.5 ± 8.85	77.3 ± 9.95	<0.001
Vegetable lipids (g)	18.9 ± 4.4	57.8 ± 5.3	<0.001
Animal lipids (g)	23.8 ± 8.3	31.4 ± 7.6	<0.05
Fish (daily servings)	0.7 ± 0.32	0.9 ± 0.41	ns
Carbohidrates (g)	164.8 ± 36.9	304.8 ± 48.3	<0.001
Fiber (g)	0.75 ± 0.35	28.9 ± 0.56	<0.001
Retinol (ug)	118.9 ± 41.3	499.8 ± 43.4	<0.001
Niacin (mg)	12.8 ± 3.8	14.7 ± 4.8	ns
Vitamin E (mg)	9.9 ± 4.6	8.8 ± 2.9	ns
Cholesterol (mg)	264.4 ± 88.9	255.3 ± 93.4	ns

P-Value <0.05 is statistically significant.

HD: hemodialysis. Ns = non significant.

Plasma triglycerides, total cholesterol and LDL-cholesterol were significantly higher (p < 0.0001, p < 0.05 and p < 0.05 respectively) while HDL-cholesterol was significantly lower (p < 0.05) in uremic patients compared to control subjects.

Plasma composition in phospholipids and fatty acids in HD patients (n = 99) and in healthy subjects (n = 160) is shown in Table 4. Several statistically significant differences are observed in HD patients compared to healthy subjects: plasma linoleic acid, dihomo- γ-linolenic acid , arachidonic acid and eicosapentaenoic acid are significantly reduced in HD patients (p < 0.0001, p < 0.0001, p < 0.0001 and p = 0.0081 respectively), while plasma γ-linolenic acid is significantly increased in the uremic patients (p < 0.0001). Finally, we did not observe between the two groups significant differences between plasma α-linolenic acid (p = 0.4460) and docosahexaenoic acid (p = 0.4389). In addition, the ω-6/ω-3 PUFAs ratios were calculated (ARA/EPA and ARA/DHA). We did not observe any significant difference in the ARA/DHA ratios between the two groups (Table 4) but we observed statistically significant difference in ARA/EPA ratios between HD patients and healthy subjects (Table 4).This last difference was not confirmed after adjustment for covariates possibly affecting plasma PUFAs concentration in HD patients and healthy controls, such as age, gender, cigarette smoking and BMI (Table 4).

**Table 4:** Plasma phospholipids

**Fatty acids (μmol/L)**	**HD (n = 99)**	**Controls (n = 160)**	**p-value**	p*
**Polyunsatured ω-6:**				
Linoleic acid (18:2)	1387.30 ± 340.15	1757.48 ± 289.93	<0.0001	<0.0001
Γ-Linolenic acid (18:3)	29.20 ± 14.83	14.79 ± 9.72	<0.0001	<0.0001
Dihomo-γ-linolenic acid (20:3)	110.68 ± 38.99	175.37 ± 65.68	<0.0001	<0.0001
Arachidonic acid (20:4)	437 ± 111.45	504.52 ± 133	<0.0001	<0.0001
**Polyunsatured ω-3:**				
Α-Linolenic acid (18:3)	18.35 ± 14.57	15.58 ± 9.62	0.4460	0.069
Eicosapentaenoic acid (20:5)	28.95 ± 37.49	39.95 ± 54.82	0.0081	0.016
Docosahexaenoic acid (22:6)	142.10 ± 67.86	150.51 ± 78.13	0.4389	0.508
**ω-6/ω-3:**				
ARA/EPA	28.54 ± 20	25 ± 17	0.0081	0.136
ARA/DHA	4.06 ± 1.53	3.48 ± 1.48	0.068	0.106
**Ω-3 Index:**				
EPA + DHA	179.46 ± 110.92	182.11 ± 111.96	0.832	0.294

P <0.05 is statistically significant, p* = differences between the two groups controlled for gender, age, BMI and smoke.

HD: hemodialysis. ARA: arachidonic acid. EPA: eicosapentaenoic acid. DHA=: docosahexaenoic acid.

Instead, for all other variables the statistical significances detected were confirmed even after adjustment for covariates previously described (Table 4).

Erythrocyte membrane composition in phospholipids and fatty acids in HD patients (n = 99) and in healthy subjects (n = 160) is shown in Table 5 as well. In particular, we observed that plasma linoleic acid (p < 0.0001), dihomo-γ-linolenic acid (p <0.0001), arachidonic acid (p < 0.0001) and α-linolenic acid (p < 0.0001) was significantly reduced in HD patients compared to controls, while plasma γ-linolenic acid (p < 0.0001) was significantly increased. On the other hand, plasma eicosapentaenoic acid (p = 0.0907) and docosahexaenoic acid (p = 0.0651) was not statistically different between the two groups, whereas the erythrocyte PUFAs ratios, ARA/EPA (p < 0.0001) and ARA/DHA (p < 0.0001), were significantly decreased in HD patients compared to healthy subjects (Table 5). Finally, we observed that the two groups had a comparable ω-3 index (EPA + DHA). Multivariate analysis, matched for age, gender, cigarette smoking and BMI, confirmed the differences observed.

**Table 5:** Erythrocyte phospholipids

**Fatty acids (μmol/L)**	**HD (n = 99)**	**Controls (n = 160)**	**p-value**	p*
**Polyunsatured ω-6:**				
Linoleic acid (18:2)	398.83 ± 119.03	684.04 ± 133.4	<0.0001	<0.0001
Γ- Linolenic acid (18:3)	1.73 ± 1.09	0.99 ± 0.42	<0.0001	<0.0001
Dihomo-γ-linolenic acid (20:3)	45.56 ± 21.14	86.94 ± 35.57	<0.0001	<0.0001
Arachidonic acid (20:4)	455.66 ± 236.66	560.37 ± 97.66	<0.0001	0.018
**Polyunsatured ω-3:**				
Α-Linolenic acid (18:3)	0.71 ± 0.4	1.02 ± 0.3	<0.0001	<0.0001
Eicosapentaenoic acid (20:5)	9.74 ± 11.8	7.43 ± 11.47	0.0907	0.334
Docosahexaenoic acid (22:6)	132.44 ± 81.39	136.48 ± 42.51	0.0651	0.001
**ω -6/ω-3:**				
ARA/EPA	83.92 ± 51.43	133.16 ± 74.62	<0.0001	0.002
ARA/DHA	4.13 ± 4.4	4.48 ± 1.62	<0.0001	0.002
**Ω-3 Index:**				
EPA + DHA	192.19 ± 139.73	143.95 ± 50.10	0.0542	0.876

P <0.05 is statistically significant p* = differences between the two groups controlled for gender, age, BMI and smoke.

HD: hemodialysis. ARA: arachidonic acid. EPA: eicosapentaenoic acid. DHA=: docosahexaenoic acid.

## Discussion

Chronic Kidney Disease (CKD) patients show lipid and lipoprotein metabolism disturbances [7]. Phosphatidylinositol and phosphatidic acid are not present in the erythrocyte membranes of CKD patients. Rheological properties of erythrocytes, such as osmotic resistance, deformability and mechanical fragility change in these patients. Many authors associate these disturbances with the lipid composition of the erythrocyte membrane [12]. During hemodialysis total cholesterol content and the cholesterol/phospholipids ratio increase in the cytoplasmic membrane. These are the main factors responsible for the alterations of membrane fluidity that potentially impact on erythrocyte deformability and osmotic resistance [13].

An abnormal phospholipid metabolism was suggested to play an important role in the progression of atherosclerosis in hemodialysis patients [14].

A number of observational studies reported an inverse association between CVD and dietary intake or plasma concentrations of omega-3 PUFAs, primarily eicosapentaenoic acid (EPA) and docosahexaenoic acid (DHA), suggesting that supplementation of ω-3 fatty acid might exert protective effects on CVD [15-19]. The protective action of ω-3 fatty acids upon the CV system is mediated by their ability to suppress inflammation and to inhibit platelet activation/adhesion, as well as by a direct effect on the myocardium [20]. This latter effect is related to their capacity to prolong the refractory state of cardiomyocytes via an interaction with fast-acting sodium channels and the L-type calcium channels. These actions are mediated by EPA and DHA in cardiomyocyte membranes. A comparative study revealed a strong correlation between n-3 fatty acids concentration in myocardium and in erythrocytes [21]. ESRD patients on maintenance HD are at risk for inadequate ω-3 intake because they may find foodstuffs less palatable as a result of alterations in taste; moreover, renal dietary recommendations do not encourage fish consumption due to adverse hyperphosphatemia [22]. Therefore, malnutrition is a frequent occurrence in HD patients and may be caused by either inadequate food intake and enhanced intradialytic loss of nutrients, or by hypercatabolism due to ESRD comorbidities or associated to dialysis treatment [23].

Phospholipids play an essential role in cell membrane structure and function. The length and degree of phospholipids and unsaturated fatty acids are main determinants of cell membrane fluidity, transport systems, activity of membrane-bound enzymes and susceptibility to lipid peroxidation [24]. The serum fatty acid lipid profile (especially phospholipids) reflects the fatty acid composition of cell membranes [25]. In our study, HD patients had reduced plasma EPA, ARA, LA and Dihomo-γ-linolenic acid (DGLA), associated with high plasma triglycerids.

Christensen and Coworkers demonstrated a possible anti-arrhythmic effect of ω-3 PUFAs, as they found a positive correlation between Heart Rate Variability (HRV) and the composition in cell membrane of ω-3 PUFAs in CKD patients [26]. Further intervention studies based on the administration of ω-3 confirmed the ω-3 PUFAs cardio-protective role [27,28] and showed that the composition of erythrocyte EPA and DHA reflects that of the myocardium and is influenced by diet [29]. We thus investigated erythrocyte membrane EPA and DHA as its “ω 3-index” (EPA + DHA) correlates to human myocardiocyte membrane ω 3-index.

We found a significant reduction of erythrocyte ω-3 fatty acid α-Linoleic Acid in HD patients compared to healthy controls. This reduction may be due to the conversion of ALA into EPA and possibly into DHA. The conversion of ALA into EPA is limited in humans, but may be physiologically and clinically important. Several dietary factors are thought to influence the conversion of ALA into EPA, the kind of protein consumed and the amount of dietary EPA among others [20]. Another possible reason for lower erythrocyte ALA in HD patients is that many foods rich in ALA (such as nuts, seeds and seed oils including flaxseed oil, English walnuts, canola oil, and soybean oil) are not recommended in uremic patients because they are rich in potassium.

Furthermore, HD patients exhibited a significant decrease of erythrocyte membrane LA and ARA that reduces the cardiovascular protective effect exerted by LA, as reported by Laaksonen et al. [30]. Arachidonic acid, a ω-6 fatty acid, is a substrate for the production of various pro-oxidant pro-inflammatory mediators and signal transduction molecules.

In previous investigations a significant increase of erythrocyte membrane fatty acids were reported in Korean ESRD patients [31]. The reason for these discrepant data is unclear. However, differences in dietary composition, genetic factors [32] and different analytical methods that have been standardized only recently may be advocated.

We finally observed both plasma and erythrocyte ω6/ω3 ratios increased in HD patients compared to healthy controls; in earlier prospective studies the supplementation of ω3 fatty acid reduced membrane ω6/ω3 ratio significantly in these patients and lowered plasma ARA in healthy subjects and in patients with coronary heart disease [33,34]. The CV protective effect of ω-3 PUFAs may be related to their anti-thrombotic, lipid-lowering and anti-inflammatory actions [35,36].

### Limitations

The main limitation of our study was a correct evaluation of the real feeding habits of HD patients on “free” diet by means of a self-administered questionnaire. A significant discrepancy is patent when the mean caloric intake reported by patients is compared to their “real” caloric intake derived from BMI calculation (see Table 3, lines 1 to 3). This underestimation of the reported caloric intake may be due to a low adherence of patients to dietary prescriptions and subsequent insincere answers.

## Conclusions

Ω-3 PUFAs may reduce this risk in the general population. A dietary treatment addressed to increase plasma ω-3 PUFAs EPA and DHA and to optimize ω-6/ω-3 ratio may exert a protective action and reduce the risk of CVD also in HD patients. The fatty acid composition of erythrocyte cell membrane reflects that of the myocardiocyte and is influenced by diet. Our study suggests that the analysis of PUFAs in plasma and erythrocyte membranes could help to identify subjects who might take advantage from a change in the dietary intake addressed to optimize the ω-6/ω-3 ratio.

## Abbreviations

CVD: Cardiovascular disease; PUFAs: Polyunsaturated fatty acids; HD: Hemodialysis; CV: Cardiovascular; CKD: Chronic kidney disease; LA: Linoleic acid; EPA: Eicosapentaenoic acid; DHA: Docosahexaenoic acid; ARA: Arachidonic acid; ALA: α-linoleic acid; DGLA: Dihomo-γ-linolenic acid; ESRD: End-stage renal disease; BMI: Body mass index.

## Competing interests

The Authors hereby certify that there is no conflict of interest with any financial organization regarding the material discussed in the manuscript or in the conclusions, implications or opinions stated.

## Authors’ contribution

MD, AN, SB designed the research and wrote the paper. PB, GN, ADS performed laboratory analysis. SR analyzed data. SMDV revised the manuscript. NDD had primary responsibility for the final content. All authors read and approved the final manuscript.
